# Prevalence, prognosis, and health care resource utilization in carriers of pathogenic germline variants in BRCA1/2 with incident early-stage breast cancer: a Finnish population-based study

**DOI:** 10.2340/1651-226X.2024.40829

**Published:** 2024-09-25

**Authors:** Peeter Karihtala, Outi Laatikainen, Samuli Tuominen, Trude Ågesen, Rasmus Eliassen

**Affiliations:** a Department of Oncology, Helsinki University Hospital Comprehensive Cancer Center and University of Helsinki, Helsinki, Finland; b Medaffcon Oy, Espoo, Finland; c AstraZeneca Nordic, Oslo, Norway; d AstraZeneca Nordic, Copenhagen, Denmark

**Keywords:** BRCA, genetics, health care resource utilization, hereditary breast cancer, prevalence, survival

## Abstract

**Background and purpose:**

Data on real-world prevalence and outcomes in patients diagnosed with pathogenic germline variants in BRCA1 or BRCA2 (gBRCAm) breast cancer is sparse.

**Material and methods:**

An observational cohort study including all patients diagnosed with incident early-stage breast cancer and recorded in Helsinki University Hospital data lake 2012–2022, accounting for one-third of the Finnish breast cancer patient population.

**Results:**

Among 14,696 incident early-stage breast cancer patients, 11.2% (*n* = 1,644) were tested for gBRCAm. Of the tested population, 7.4% (*n* = 122) carried gBRCAm. Of the 122 gBRCAm patients, 95.1% (*n* = 116) were women, with a median age at diagnosis of 46.4 years. Among the same patient group, HER2 status was available for 87.7% (*n* = 107) of the patients. Among these, 49.5% (*n* = 53) had hormone receptor-positive (HR+), HER2-negative breast cancer, 13.1% were (*n* = 14) HER2-positive, and 37.3% (*n* = 40) of patients had triple-negative breast cancer. The tested patients were significantly younger compared with non-tested patients. No significant differences in overall survival or healthcare resource utilization between the tested patients with gBRCAm and gBRCA wild-type (gBRCAwt) were observed.

**Interpretation:**

This comprehensive observational study supports previous findings of gBRCAm prevalence in the Western early-stage breast cancer population. While no differences in survival were observed between patients with gBRCAm and gBRCAwt, it is important to consider the potential influence of selection bias, particularly due to the younger gBRCAm testing target population and the overall low frequency of testing. Therefore, a substantial proportion of the patients carrying gBRCAm likely remained undiagnosed, and wider screening criteria are warranted.

## Introduction

Germline pathogenic variants in *BRCA1* and *BRCA2* genes (gBRCAm) are the most common hereditary breast cancer predisposition [1]. So far, identifying gBRCAm has mainly offered information on cancer risks, notably the risk of breast cancer, with a lifetime risk of 50–70% for patients with pathogenic variants in *BRCA1* [2, 3]. Consequently, the information on gBRCAm status has been used as a guidance in the selection of risk-reducing surgery [4, 5]. The oncological landscape to treat breast cancer patients with gBRCA-mutations has been changed after tpoly (ADP-ribose) polymerase (PARP) inhibitors improving progression-free survival in the treatment of metastatic breast cancer and, recently, also improvement in overall survival in the early-stage breast cancer setting [6–8]. This has been one factor leading to recent wider recommendations to screen for gBRCAm in patients with breast cancer [9–11].

There is conflicting evidence regarding the prognostic value of gBRCAm, although several studies suggest worse OS for gBRCAm breast cancer patients. Most studies, however, are limited by patient selection, small sample sizes and analyses impacted by other prognostic factors such as age, tumor/nodal status, grade, hormone receptor (HR) status, year of diagnosis, oophorectomy and use of chemotherapy, which are often unevenly distributed between gBRCAm-carriers and non-carriers. [12–16]. The prospective POSH study reported a better overall survival (OS) for TNBC gBRCAm versus TNBC gBRCAwt at 2 years, but at 5 and 10 years, no difference was observed. In the overall gBRCAm group, irrespective of biological subgroup, no difference in survival was observed [12]. Another study reported no difference in breast cancer specific survival for TNBC gBRCAm versus gBRCAwt, but a worse breast cancer specific survival for estrogen receptor positive gBRCAm versus gBRCAwt. Overall, there was a marginally significant trend for worse breast cancer specific survival for gBRCAm versus gBRCAwt [13].

Thus, assessing the gBRCAm status is becoming increasingly important in the treatment selections of early-stage breast cancer. Therefore, this registry-based study aimed to explore the prevalence of gBRCAm among patients with incident early-stage breast cancer and describe their characteristics based on a data lake covering the specialized care of more than two million Finnish residents. Healthcare resource utilization (HCRU) was also evaluated due to very limited knowledge of HCRU use in patients with early-stage breast cancer carrying gBRCAm.

## Materials and methods

### Study design and population

This observational population-based study included all individuals aged ≥ 18 years at the first recorded diagnosis of breast cancer (i.e., the index date), as identified from the inpatient electronic medical records (EMRs) of Helsinki University Hospital (HUS) Data Lake between 1^st^ January 2012 and 31^st^ May 2022. Patients were followed up from the index date until death or the end of the study period (November 30^th^, 2022), allowing for a minimum follow-up period of 6 months. Only patients resident at HUS catchment area at data extraction were included in the study. The HUS data lake covers almost 50% of the annual breast cancer cases in Finland, as HUS provides specialized healthcare services for 2.2 million patients in Southern Finland and is almost solely publicly funded. For the identified breast cancer cohort, data on all diagnoses and procedures were collected from 2005 and onwards, while data on patient demographics and clinical factors, hospital-administered medications, treatments, laboratory, radiology and pathology results, and deaths were available from 2012. Helsinki University Hospital granted permission for the study (HUS/224/2022) by the provision of the Act on the Secondary Use of Health and Social Data (552/2019), without the need for informed consent due to the registry-based nature of the study.

### Exclusion criteria

Only individuals with their first breast cancer diagnosis (i.e., new incident diagnoses) recorded during the study period (2012–2022) were eligible for inclusion, excluding potential prevalent breast cancers (i.e., patients diagnosed before the start of the study period), allowing for a minimum washout period of 8 years (Supplementary Figure 1). Individuals with a home municipality outside of the HUS region were excluded to ensure the availability of follow-up data in the HUS data lake. Patients who had initiated CDK4/6i treatments before the treatment and became reimbursable were excluded, as they were likely part of a trial. Patients with statements positive for gBRCA in the pathology reports specified as non-pathogenic or ‘variant of unknown significance’ were excluded from the gBRCAm cohort.

### Exposure ascertainment

The EMRs of the HUS data lake were used to extract data on breast cancers based on the International Classification of Diseases 10^th^ edition (ICD-10) code C50 (Supplementary Figures 1–2). To differentiate early-stage breast cancer patients from metastatic breast cancer patients, individuals with metastasis diagnosed at most 90 days after the index date or during adjuvant chemotherapy were considered having *de novo* advanced breast cancer (*de novo* aBC). Metastases were detected based on the following criteria: (1) ICD-10 codes C78 or C79 recorded or AJCC stage IV or TNM-stage M1 found in the EMRs; (2) aBC specific treatments with procedure code WD* (‘treatment of metastasis’) or radiotherapy of advanced disease based on procedure codes indicating aBC (recorded ≥ 2 times, applying the latest recorded date), or patients who had received aBC specific treatment (i.e., fulvestrant, palbociclib, abemaciclib, ribociclib, eribuline, gemcitabine or lapatinib); or (3) applying text mining with the criteria of ≥ 2 mentions of metastasis in the radiological statements or general patient texts with two records at most 6 months apart.

Patients with early-stage breast cancer, but without NGS panel test for *BRCA1/2* were defined as non-tested patients with early-stage breast cancer. Patients tested for only sporadic *BRCA1/2* pathogenic variants were excluded from the analyses. gBRCA pathogenic variant statuses of the gBRCA1/2-tested patients were obtained by text mining test results available as free text in the pathology and laboratory records. Patients who tested negative for gBRCA1/2 pathogenic variants were defined as patients with ‘gBRCA1/2wt’. Patients who tested positive for non-pathogenic gBRCA1/2 variants or with gBRCA1/2m of unknown significance were excluded from further analyses. Patients who tested positive for pathogenic gBRCAm were defined as ‘gBRCA1/2m’ patients.

For most of the inclusion period, the main testing criteria for breast cancer hereditary disposition were: (1) at least three breast or ovarian cancers in the first or second-degree relatives and at least one of these in the age of under 50 years old; (2) two breast cancer cases in the first or second-degree relatives, both under 45 years old; (3) breast cancer at the age of under 40 years old; (4) TNBC or medullary breast cancer under the age of 60 years old; (5) bilateral breast cancer, when both diagnosed at the age of under 40 years old; (6) breast cancer under 50 years old and ovarian cancer in the first or second-degree relative; (7) breast and ovarian cancer in the same individual; (8) male breast cancer.

### Biological subtypes

Patients were further stratified according to the biological subtype based on hormone receptor (HR) and HER2 status, categorized as (1) HER2-positive; (2) HR-positive and HER2-negative; and (3) triple-negative breast cancer (TNBC), and (4) unknown. The stage was categorized as I-IV, according to the TNM classification of malignant tumors.

Patients with either HER2 immunohistochemistry (IHC) of 3+, positive in situ hybridization (ISH) result or patients who received anti-HER2 treatment were considered as HER2-positive. HR-positive breast cancer was defined as positive with estrogen receptor (ER) and/or progesterone receptor (PR) immunopositivity in 1–100% of tumor cells, or if the patient had received aromatase inhibitor or tamoxifen ascertained based on the WHO’s Anatomical Therapeutic Chemical (ATC) code classification. The criteria for both HER2 and HR status were evaluated within 90 days before and 365 days after the breast cancer diagnosis. In case of multiple, discordant IHC/ISH or HR staining results, the most recent was used.

### Outcome ascertainment

The survival endpoints for OS, invasive disease-free survival (IDFS) and distant-disease-free survival (DDFS) were ascertained from the EMRs of the HUS data lake based on an *a priori* set definitions. In brief, OS was defined as the time from the breast cancer diagnosis until the date of death (all-cause). IDFS (including per definition all events under DDFS) was defined as the time from the breast cancer diagnosis (the first recorded date) until one of the following: (1) distant recurrence (detection of metastasis, coincides with the definition of metastatic breast cancer); (2) second primary invasive cancer; (3) death from any cause; (4) chemotherapy or radiotherapy re-initiation after 1 year time period from the end of adjuvant treatment; or (5) invasive breast cancer diagnosis in the pathology records 6 months after adjuvant treatment. DDFS was defined as the time from the breast cancer diagnosis until (1) distant recurrence (detection of metastasis, coinciding with the definition of metastatic breast cancer) or (2) death from any cause.

### Ascertainment of HCRU and related costs

For the assessment of HCRU and related costing estimates, both all-cause and breast cancer-related outcomes were assessed based on specialized care contacts, diagnoses, duration of hospitalizations and contact types (in- and out-patient care), as ascertained from the EMRs of the HUS data lake. The assessed variables included the number of in-hospital admissions, length of in-hospital stay (LOS) and the number of outpatient contacts (e.g., calls, digital visits, physical visits, and treatment-related visits).

### Statistical analyses

Summary statistics for patient demographics and cancer-related characteristics were presented as mean/median, standard deviation (SD)/interquartile range (IQR), number of patients and proportions as appropriate. Differences in proportions between categorical variables were tested with the two-sided Chi-squared test, and continuous variables were tested with Mann–Whitney U-test. Charlson comorbidity index (CCI) score was treated as a binary variable (i.e., score 0 and ≥ 1). Patients were sub-grouped as early-stage and *de novo* advanced breast cancer, and by the germline BRCA1/2 mutation status (gBRCAm-tested and non-tested patients). *P*-values less than 0.05 were considered indicative of statistical significance. No adjustment for multiple testing was performed. Whereas missing values were not imputed, the proportion of missing values was reported (as applicable). Due to the Finnish legislation, presenting the exact number of patients was prohibited, when the size of the sub-cohort was between 1 and 4.

OS, IDFS, and DDFS were estimated using the Kaplan–Meier method. The survival curves were drawn with the 95% confidence intervals (CI; type: log-log confidence intervals). Multivariable Cox proportional hazard models were used to test the difference in OS and DDFS between patients with gBRCAm and gBRCAwt. All outcome analyses were censored at the end of the study period. The proportional hazards assumption was tested and evaluated visually by plotting Schoenfeld residuals (data not shown). The covariates included in the analyses were age, sex, the year of breast cancer diagnosis, and biological subtype. The hazard ratios (HR) with 95% CIs and *p*-values were reported using forest plots, and the analyses were sub-grouped by the germline BRCA1/2 mutation status and biological subtype.

The HCRU and associated cost estimates were derived using the unit cost method, where the average cost of each hospitalization and outpatient contact was added based on the specialty of the contact. Prices were obtained from Mäklin et al., which contained the average cost of all diagnosis and treatment-related costs, including medications and personnel fees per hospital day or outpatient contact of each specialty [17]. The breast cancer-specific HCRU-related costs were assessed based on the diagnosis codes recorded in conjunction with the contacts to public healthcare.

In all HCRU analyses, patients were followed up from the first recorded breast cancer diagnosis up until a possible diagnosis of metastatic breast, death, or end of the study period. Thus, the results reflect the treatment of early-stage breast cancer, excluding any potential cost after metastatic onset. Per patient year (PPY) estimates were reported, which were defined as total events or costs divided by the total follow-up length obtained by summing over the patients. Resource use (number of events) and related costs (€) of days at hospital/hospitalizations, outpatient visits and radiotherapy visits were reported separately. The 95% CIs for the HCRU analyses were estimated with bootstrap sampling (*10,000* samples) due to skewness and non-normal distribution of resource and cost variables.

All analyses were performed on R, a software for statistical computing (version 4.0.3).

## Results

**Figure 1:**
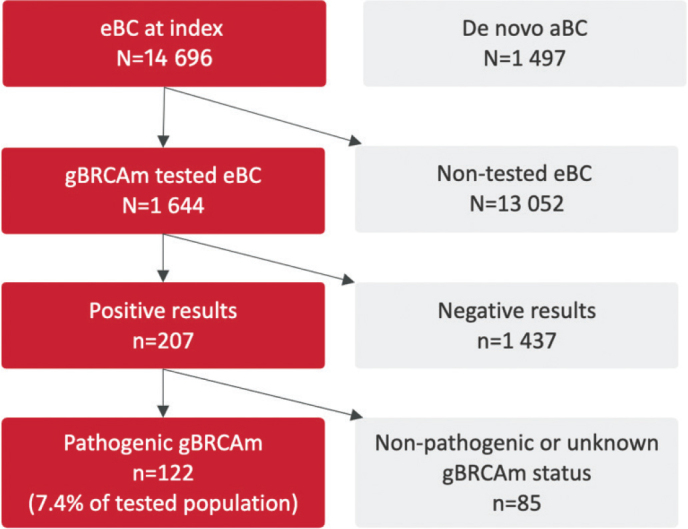
Flowchart of the tested and non-tested patients with early-stage breast cancer.

The results of this study are reported in accordance with the Strengthening the Reporting of Observational Studies in Epidemiology (STROBE) statement guidelines [18]. A total of 14,696 early-stage breast cancer patients were identified from 1st January 2012 to the end of follow-up time, 30th November 2022, and for 11.2% (*n* = 1,644) of the patients, gBRCAm testing results were available (Figure 1). Of the 1,644 tested patients, 1,437 results were categorized as unsuspicious and 207 with a suspicious gBRCAm statement in the pathology reports. Of these, 74 results were defined as non-pathogenic, 11 with variants of unknown significance (VUS) and 122 identified with a pathogenic variant of *BRCA1* or *BRCA2* (gBRCAm) corresponding to 7.4% of the tested population.

Compared to the non-tested population, the proportion of younger patients (aged < 45 years) and males was higher among gBRCAm-tested patients (Table 1). The median age for gBRCAm-tested patients at diagnosis was 49.6 years (interquartile range (IQR) 39.8 – 60.5), and the mean age was 51.1 years (SD 13.7). For non-tested, the median age was 66.2 years (IQR 57.3 – 74.4), and the mean age was 65.8 years (SD 12.4).

**Table 1:** Demographics and cancer-related characteristics of early-stage and advanced breast cancer patients in both pathogenic germline BRCA1/2 mutation (gBRCAm) tested and non-tested patients.

Variable	Level	gBRCAm tested		Non-gBRCAm tested		*P*-value
		Number of patients	%	Number of patients	%	
		*n* = 1,644	11.2	*n* = 13,052	88.8
Age at breast cancer diagnosis (years)	Median (IQR)	50.8 (39.8–60.2)		65.6 (56.8–74.1)		<0.001
Length of follow-up (years)	Median (IQR)	4.0 (2.2–6.7)		5.0 (2.5–7.9)		-
	Mean (SD)	4.6 (2.9)		5.2 (3.1)		<0.001
Age group (years) at breast cancer diagnosis	Less than 40	419	25.2	155	1.2	<0.001
	40–44	212	12.9	362	2.8	
	45–54	435	26.5	2 234	17.1	
	55–64	285	17.3	3 506	26.9	
	≥ 65	293	17.8	6 795	52.1	
Sex	Female	1,608	97.8	13,002	99.6	<0.001
	Male*	36	2.2	50	0.4	
Year of breast cancer diagnosis	2012–2013	183	11.1	2,715	20.8	<0.001
	2014–2015	246	15.0	2,555	19.6	
	2016–2017	300	18.2	2,300	17.6	
	2018–2019	391	23.8	2,360	18.1	
	2020–2021	430	26.2	2,554	19.6	
	2022**	94	5.7	568	4.4	

Dx: diagnosis; IQR: interquartile range.

* Percentages calculated from all gBRCA tested/non-gBRCA tested male patients.

** The patients were identified up until 31^st^ May 2022 and followed-up until 30^th^ November 2022.

Patient and disease characteristics for patients with early-stage gBRCAm-positive and gBRCAm-negative cancer are presented in Table 2. A higher frequency of TNBC cases was observed in gBRCAm patients (32.8%) compared with the gBRCAwt patients (19.4%). In the final cohort of 122 gBRCAm-positive patients, HER2 status was available for 87.7% (*n* = 107) patients. Out of these, 11.5% (*n* = 14) were classified as HER2 positive, 43.4% (*n* = 53) as HR-positive/HER2 negative, and 32.8% (*n* = 40) as TNBC. Characteristics of gBRCAm-positive patients according to the biological subgroup are shown in Supplementary Table 1.

**Table 2:** Demographics and tumor characteristics of patients with early breast cancer according to pathogenic germline BRCA1/2 mutation (gBRCAm) status.

Variable	Level	gBRCAm	gBRCAwt	*P*-value
		Number of patients (%)	Number of patients (%)	
		122 (100%)	1,456 (100%)	
Sex	Female	116 (95.1%)	1,428 (98.1%)	0.062
	Male	6 (4.9%)	28 (1.9%)	
Age (years) at breast cancer diagnosis	Median (IQR)	46.4 (38.1–55.8)	49.3 (39.9–60.2)	0.099
Age group (years) at breast cancer diagnosis	Less than 40	37 (30.3%)	370 (25.4%)	0.4498
	40–44	19 (15.6%)	197 (12.8.3%)	
	45–54	33 (27%)	389 (26.7%)	
	55–64	15 (12.3%)	254 (17.4%)	
	≥65	18 (14.8%)	256 (17.6%)	
Biological subtype	HER2+	14 (11.5%)	263 (18.1%)	0.0018
	HR+ and HER2-	53 (43.4%)	765 (52.5%)	
	TNBC	40 (32.8%)	283 (19.4%)	
	Unknown	15 (12.3%)	145 (10.0%)	
Tumor size^6^	At most 2 cm	52 (56.5%)	685 (58.1%)	0.860
	More than 2 cm	40 (43.5%)	495 (41.9%)	
Number of positive lymph nodes^1^	0	41 (48.8%)	545 (46.6%)	0.963
	1–3	28 (33.3%)	404 (34.6%)	
	4+	15 (17.9%)	220 (18.8%)	
Stage^2^	I	24 (32%)	354 (32.6%)	0.927
	II	35 (46.7%)	483 (44.5%)	
	III	16 (21.3%)	248 (22.9%)	
Grade^3^	1–2	68 (71.6%)	655 (55%)	0.003
	3	27 (28.4%)	536 (45%)	
IHC score (HER2)^4^	0	26 (31.7%)	266 (27.6%)	0.004

Most of the patients received adjuvant therapies (chemotherapy 76.2%), followed by neoadjuvant chemotherapy (23.8%) (Table 3). More than half of the patients (59.0%) received radiotherapy. In the biological subgroup of ‘unknown’ there was missing breast surgery data for 46.7% of the patients.

**Table 3:** The surgical and (neo)adjuvant treatments of the patients carrying pathogenic germline BRCA1/2 mutation (gBRCAm) and early breast cancer.

Treatment type	All, *N* = 122	HER2+, *n* = 14	HR+ and HER2-, *n* = 53	TNBC, *n* = 40	Unknown, *n* = 15
	N (%)	N (%)	N (%)	N (%)	N (%)
Surgical operation	115 (94.3%)*	14 (100%)	53 (100%)	40 (100%)	8 (53.3%)
Mastectomy**	93 (80.9%)	Censored***	43 (81.1%)	32 (80.0%)	Censored***
Lumpectomy**	22 (19.1%)	<5	10 (18.9%)	8 (20.0%)	<5
Radiotherapy	72 (59%)	8 (57.1%)	33 (62.3%)	28 (70%)	<5 (-%)
Neoadjuvant chemotherapy	29 (23.8%)	<5 (-%)	9 (17%)	14 (35%)	Censored***
Endocrine treatment	Censored***	8 (57.1%)	43 (81.1%)	0 (0%)	<5 (-%)
Anti-HER2 therapy	11 (9%)	11 (78.6%)	0 (0%)	0 (0%)	0 (0%)
Chemotherapy (in any early treatment setting)	93 (76.2%)	12 (85.7%)	37 (69.8%)	38 (92.3%)	6 (40.0%)
Adjuvant chemotherapy	Censored	10 (71.4%)	32 (60.4%)	31 (77.5%)	<5 (-%)

HER2: human epidermal growth factor receptor 2; HR: hormone receptor; TNBC: triple-negative breast cancer.

* The remaining 5.7% of patients had no records for surgery and pathological criteria (due to lack of biopsy data).

** Percentages per patients operated.

*** Per Finnish legislation, groups with patient numbers 1–4 cannot be reported, and the exact number of patients may not be inferable. Thus, at least one other (sub)group within the category must be censored to prevent retrieving the exact number of patients in all subgroups.

### Survival outcomes of patients with gBRCAm

**Figure 2:**
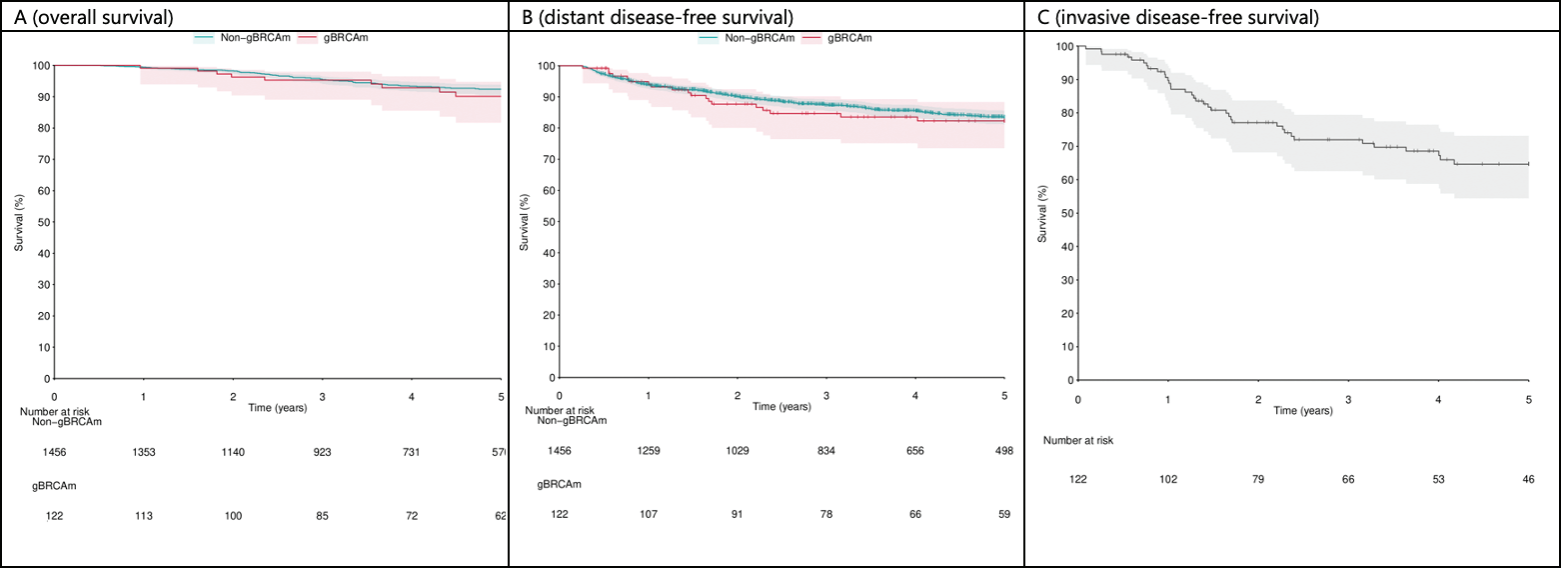
(A) Overall survival (OS), (B) distant disease-free survival (DDFS) in all pathogenic germline BRCA1/2 mutation (gBRCAm) tested patients according to the gBRCAm status (pathogenic or non-gBRCAm/negative for gBRCA1/2 mutation), and (C) invasive disease-free survival (IDFS) in patients with gBRCAm and early-stage breast cancer.

The presence of gBRCAm was not significantly associated with an improved OS (hazard ratio (HR) 0.78; 95% CI 0.24–2.53; *p* = 0.685) or DDFS compared with patients who had undergone testing but were classified as gBRCAwt (HR = 0.82; 95% CI 0.36–1.87; *p* = 0.634; Figure 2, Supplementary Figures 3–5).

Later enrolment to the study cohort was associated with poorer OS among gBRCAm-tested patients in the multivariable model (HR = 1.16; 95% CI: 1.06–1.28; *p* = 0.002; hazard ratio per 1 year; Supplementary Figure 3) adjusted by age, sex, biological subtype and gBRCAm status. The year of breast cancer diagnosis was not significantly associated with any of the other covariates. Similarly, when the start date of the follow-up was changed from the breast cancer diagnosis to the gBRCA test date, no association was found between the year of breast cancer diagnosis and OS (HR = 0.97, 95% CI: 0.89–1.04, *p* = 0.385; Supplementary Figure 4).

**Figure 3:**
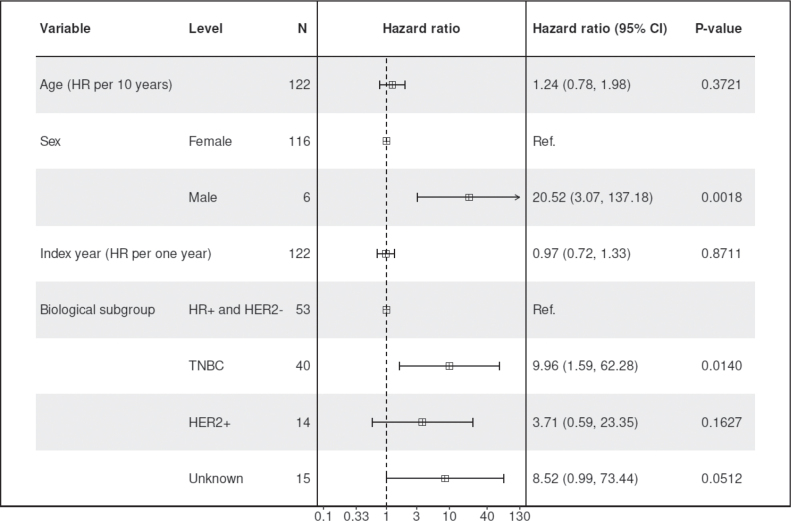
Multivariable Cox model for overall survival (OS) among patients with pathogenic germline BRCA1/2 mutation (gBRCAm) and early-stage breast cancer during the study period.

Among the patients with gBRCAm, TNBC subtype was associated with poorer OS compared to patients with HR-positive and HER2-negative breast cancer, based on a smaller sample size (HR = 9.96, 95% CI: 1.59–62.28, *p* = 0.014; Figure 3, Supplementary Figure 6). The direction of the effect was consistent for DDFS (HR = 3.51, 95% CI: 1.00–12.40, *p* = 0.051; Supplementary Figure 8) and IDFS (HR = 2.76, 95% CI: 1.28–5.94, *p* = 0.0097; Supplementary Figure 10).

### Healthcare resource utilization

**Figure 4:**
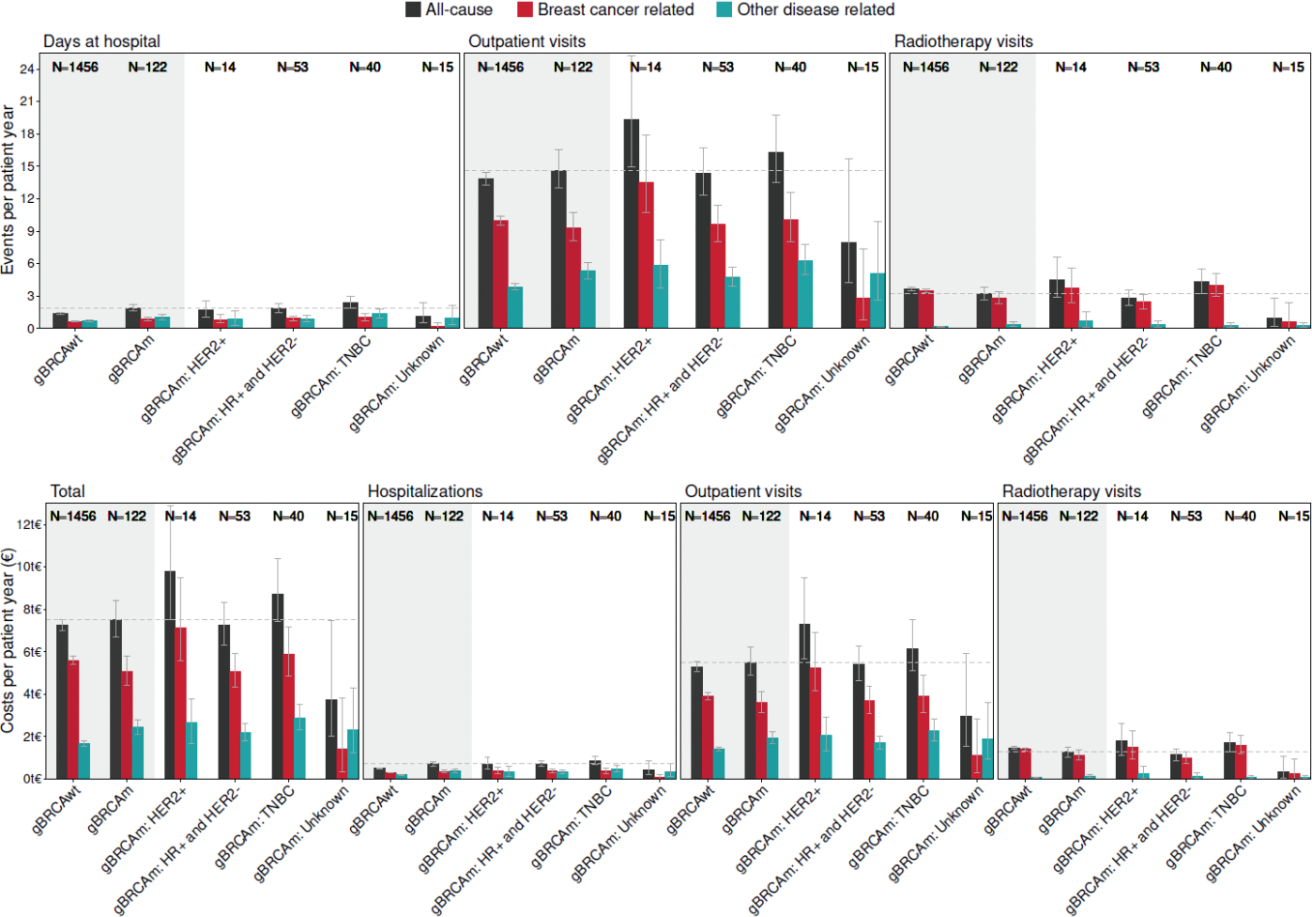
Healthcare resource utilization (HCRU) and related costs among patients with pathogenic gBRCA1/2 mutation (gBRCAm) overall, according to the biological subtype of breast cancer and among the patients without pathogenic gBRCAm.

There was no significant difference in HCRU-related costs between gBRCAm and gBRCAwt patients, with the costs being 7,505€ PPY (95% CI 6,694–8,423€ PPY) and 7,264€ PPY (95% CI 7,010–7,532€), respectively (Figure 4). Among the gBRCAm patients, those with HR-positive and HER2-negative subtypes had the lowest HCRU-related all-cause costs, 7,271€ PPY (95% CI 6,322€–8,332€), followed by the patients with TNBC, 8,724€ PPY (95% CI 7,411€–10,405€). The highest HCRU-related costs were found among gBRCAm HER2-positive breast cancer patients (9,792€ PPY, 95% CI 7,526–12,888€), yet based on a smaller group (*n* = 14). Most of the costs associated with gBRCAm patients were accumulated during the adjuvant chemotherapy and radiotherapy (Supplementary Figure 10).

## Discussion

In this population-based observational study including over 14,000 early-stage breast cancer patients and over 1,600 gBRCAm-tested patients, the overall prevalence of gBRCAm within the incident early-stage breast cancer population was 7.4% (out of the gBRCAm-tested population). Based on these numbers and the results from earlier studies focusing on gBRCAm prevalence, it is likely that a significant proportion of gBRCAm carriers with early-stage breast cancer remain undiagnosed.

gBRCAm is associated with younger age, lower stage at diagnosis and more usually triple-negative subtype. The previously reported gBRCAm prevalence rates, including both early-stage and metastatic breast cancers, have ranged between 2.7% and 6.1% in patients with mixed hormone receptor status and up to 15.4% in the TNBC-enriched population [19–22]. Only a few studies have previously focused specifically on gBRCAm in early-stage breast cancer or only on pathogenic variants of *BRCA1/2*. Gonzalez-Rivera et al. reported deleterious (not necessarily pathogenic) gBRCAm in 14% of the 105 stage II-III patients, while a single-center study by Tung et al. found deleterious gBRCAm in 10.7% of 488 studied stage I-III patients [23, 24]. In the large POSH study, including over 2,700 patients, the prevalence of gBRCA1/2 mutations was 12% in the population of women 40 years or younger [12]. To the best of our knowledge, only one study, with 20 BRCA1/2 mutation carriers, has previously reported gBRCA1/2 variants only in the early-stage breast cancer setting [22]. The patients in our study were referred to testing according to the specific criteria, which became more established in the public healthcare setting in Finland during the study period 2012–2022 and it is reflected by the significantly increased proportion of the gBRCAm tested patients in the latest period of the study. Thus, the gBRCAm prevalence of 7.4% in the tested incident early-stage breast cancer population appears to be in line with the previous yet limited literature on this topic. Still, a systematic prospective gBRCAm testing would be needed to determine the true prevalence of the carriers. Here, our data show that almost 70% of the patients were tested after receiving their breast cancer diagnosis.

Pathogenic BRCA1/2 variants have been linked to poorer OS in patients with breast cancer but without significant impact on breast cancer-specific survival [14, 15, 25]. In our cohort of 122 patients with gBRCAm, we did not find associations between gBRCAm status and survival, but the studied population was primarily selected by age and subtype, given the clinical praxis of testing individuals. However, TNBC was observed as a suggestive risk factor for lower OS (based on point estimates) in this study, although it was based on a smaller number of patients and inherently wider confidence intervals. While it has been unclear whether ER negativity is associated with worse or improved outcomes also among gBRCAm patients, the TNBC phenotype has not been previously reported as a prognostic factor in early-stage breast cancer patients with gBRCAm [13, 16, 25, 26].

Recent studies with varying incremental lifetime effects, costs, and willingness-to-pay thresholds, have concluded that screening for gBRCAm is cost-effective, both in women at high risk of breast cancer, prior to disease onset and after having breast cancer diagnosis [27–29], also considering a recent indication for adjuvant olaparib treatment [30]. There is still a notable gap in the knowledge of HCRU in early-stage breast cancer patients carrying gBRCAm. Although gBRCAm carriers have been treated similarly as sporadic cancer patients until recently [8], in theory, gBRCAm patients could have higher HCRU costs due to a more frequent magnetic resonance imaging (MRI) screening or a higher proportion of TNBC patients, requiring more often resource consuming neoadjuvant chemotherapy [31, 32]. Based on the current results, the patients with gBRCAm had equal HCRU to gBRCAwt patients. The results could have been different in populations with less risk-reducing mastectomies, which leads to the decrease in the use of MRI monitoring or in countries where neoadjuvant treatment is the mainstream treatment modality in TNBC, which was not a case in Finland during the period of this study. Also, the almost solely publicly funded healthcare in Finland is likely not to affect the results.

TNBCs covered one-third of biological subtypes in the patients with pathogenic gBRCA1/2 variants. While the biological subtype in the patients with pathogenic variants of BRCA2 resembles distribution of sporadic breast cancers to a great extent, most breast cancers in pathogenic BRCA1 variant carriers have TNBC subtype [12]. Likely due to the testing criteria, TNBCs were also overrepresented, with a proportion of one-fifth among the patients who underwent genetic testing without pathogenic findings. Most of the gBRCAm patients had an HR-positive subtype, and this has also been considered in the latest gBRCAm screening guidelines [10]. In addition, the recently updated guidelines from 2024 of the Finnish Breast Cancer Group recommend genetic testing for all new breast cancer patients under the age of 60 years in the initial treatment planning [33]. In our study, the tested patients were younger than the non-tested. Among the tested patients, there were no differences in the stage, CCI score, sex, or age distribution between those with gBRCAm1/2 variants compared with those without such variants. HER2 positivity was not uncommon among those with pathogenic gBRCA1/2 variants, and future studies could investigate the combination of HER2-targeted therapies and PARP inhibitors. Contrary to the previous studies, differentiation in the cancers of gBRCAm carriers was better than in those without pathogenic variants [34]. This could potentially derive from the more precise screening of the carriers, but again, there were no differences in tumor size and lymph node involvement between the carriers and non-carriers. In this study setting, we did not have the information about which proportion of the gBRCAm carriers were under MRI surveillance.

To the best of our knowledge, this is among the largest population-based studies to explore gBRCAm testing and the prevalence of pathogenic BRCA1/2 variants within incident early-stage breast cancer patients. The study was performed by analyzing a data lake covering specialized healthcare services for 2.2 million unique individuals. No previous studies have evaluated HCRU costs in this potentially costly population. We also acknowledge several potential limitations in this study. We did not separate *BRCA1* and *BRCA2* mutations. Both are considered high-risk genes but have differences in biology and oncological predictive relevance. Due to the observational design of the study, the EMRs were not manually searched, which may have potentially led to underdiagnosis, but likely not to the overestimation of the actual gBRCAm prevalence. The patients were selected for gBRCAm testing mainly according to the age- and subtype-based criteria, inherently introducing a selection of the tested population, and therefore limiting the generalizability of the results to similar settings and populations with similar criteria for testing. The HCRU-associated costs were based on data from public hospitals. However, as oncological treatments and surveillance have been strongly concentrated in the public sector in Finland and given almost all Finnish gBRCAm patients have their follow-up in tertiary healthcare, the presented figures are likely to reflect reality.

In conclusion, these results from a large university hospital data lake support from the few previous studies regarding the prevalence of gBRCAm among early-stage breast cancer patients. So far, this study is one of the largest and the first one to concentrate only on the gBRCAm carriers in early-stage breast cancer. There were no differences in overall survival between gBRCAm and gBRCAwt early-stage breast cancer patients and the HCRU-associated costs appear to be similar between gBRCAm and gBRCAwt early-stage breast cancer patients. However, most of the gBRCAm carriers are likely undiagnosed, given the clinically applied criteria for testing during the study period. Therefore, wider screening criteria for gBRCAm, as the recently updated Finnish guidelines, are warranted and it is imperative that guidelines are being adhered to, given the availability of novel treatments, including PARP-inhibitors specifically targeting this population.

## Supplementary Material

Prevalence, prognosis, and health care resource utilization in carriers of pathogenic germline variants in BRCA1/2 with incident early-stage breast cancer: a Finnish population-based study

## Data Availability

The dataset from this study is held at Findata’s secure operating environment, Kapseli, yet data sharing agreements prohibit making the dataset publicly available. The data and the underlying analysis plan are, however, available from the authors upon a reasonable request to the corresponding author (PK) and after obtaining the relevant permissions from Finnish Authorities.
