# Supplementary material for: Prevalence, prognosis, and health care resource utilization in carriers of pathogenic germline variants in BRCA1/2 with incident early-stage breast cancer: a Finnish population-based study
Source: Acta Oncol. 2024 Sep 25;63:40829. doi: 10.2340/1651-226X.2024.40829 (PMC11445600; doi:10.2340/1651-226X.2024.40829)
Supplement: Prevalence, prognosis, and health care resource utilization in carriers of pathogenic germline variants in BRCA1/2 with incident early-stage breast cancer: a Finnish population-based study [file AO-63-40829-s1.pdf]

Supplementary material has been published as submitted. It has not been copyedited, or typeset by Acta Oncologica

Supplementary materials

- **Supplementary Figure 1:** Flowchart of the final cohort formation

  Supplementary Figure 2: Flow chart of the data collection and cohort formation

- **Supplementary Figure 3:** Multivariable Cox proportional hazards model for the overall survival in all gBRCAm-tested patients

- **Supplementary Figure 4:** Multivariable Cox model for overall survival defined **as the time from** gBRCAm **test** (if done before breast cancer diagnosis) until death among all germline BRCA1/2 mutation tested patients

- **Supplementary Figure 5:** Multivariable Cox model for distant disease-free survival among all gBRCAm-tested patients according to gBRCA mutation status

- **Supplementary Figure 6:** Overall survival, distant disease-free survival, and invasive disease-free survival in patients with and without pathogenic gBRCAm according to the biological subgroup of breast cancer

- **Supplementary Figure 7:** Invasive disease-free survival among patients with pathogenic gBRCA1/2 mutation according to the biological subgroup of the breast cancer.

- **Supplementary Figure 8:** Multivariable Cox model for distant disease-free survival among patients with gBRCAm early-stage breast cancer

- **Supplementary Figure 9:** Multivariable Cox model for invasive disease-free survival among patients with gBRCAm early-stage breast cancer

- **Supplementary Figure 10:** Healthcare resource use and related costs in the gBRCAm patients during adjuvant treatment (time from breast cancer diagnosis until the end of adjuvant chemotherapy or radiotherapy) and during follow-up afterwards

- **Supplementary Table 1:** Patient demographics and cancer characteristics of patients with pathogenic gBRCAm and early-stage breast cancer according to the biological subgroup

- **Supplementary Table 2:** Numerical estimates at one year interval of all outcome analyses.

- **Supplementary Table 3.** Numerical estimates of the health care resource utilization and related costs per patient year among patients without pathogenic gBRCA1/2 mutation according to the biological subgroup of the breast cancer.

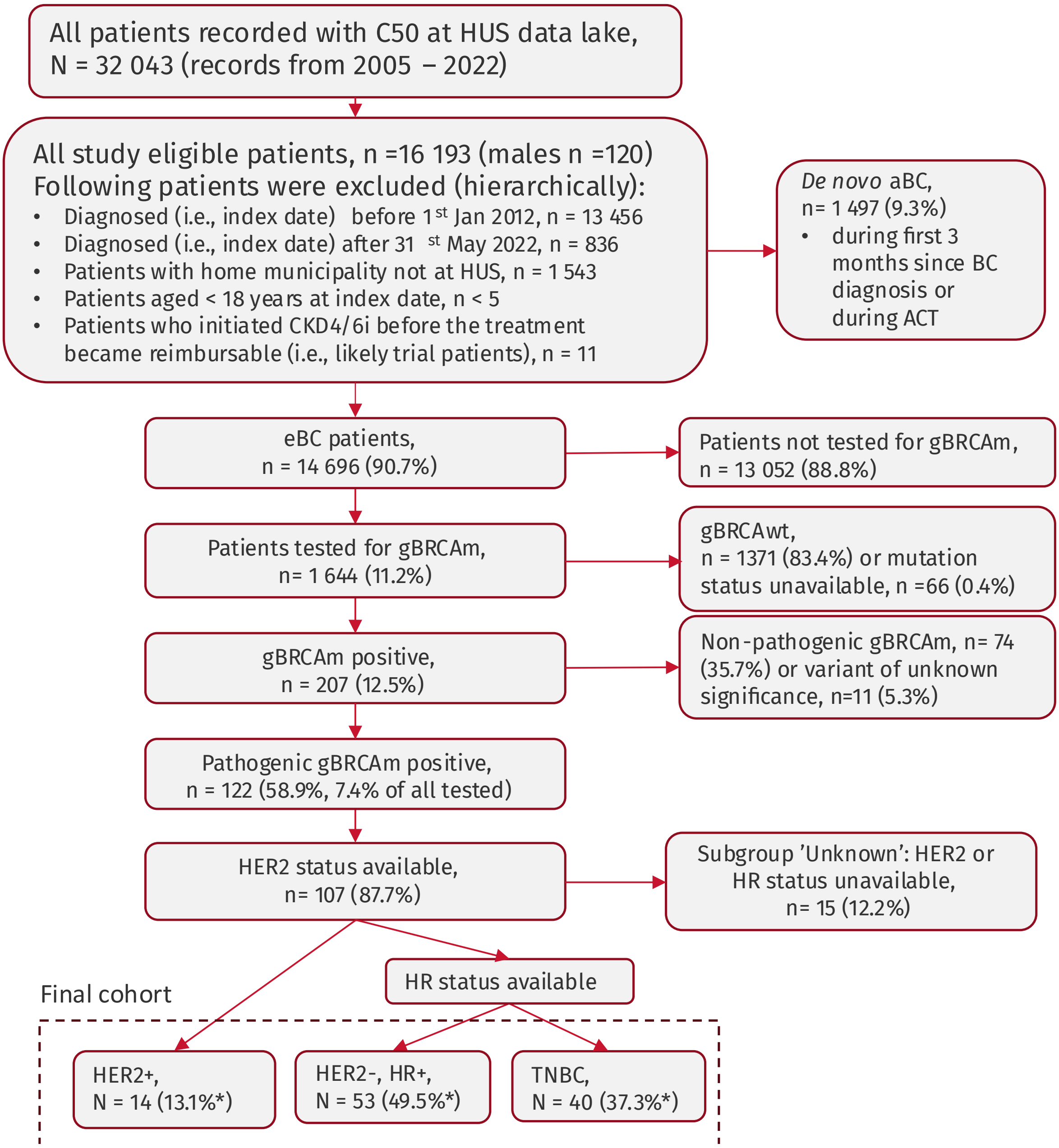

Supplementary Figure 1. Flowchart of the final cohort formation.

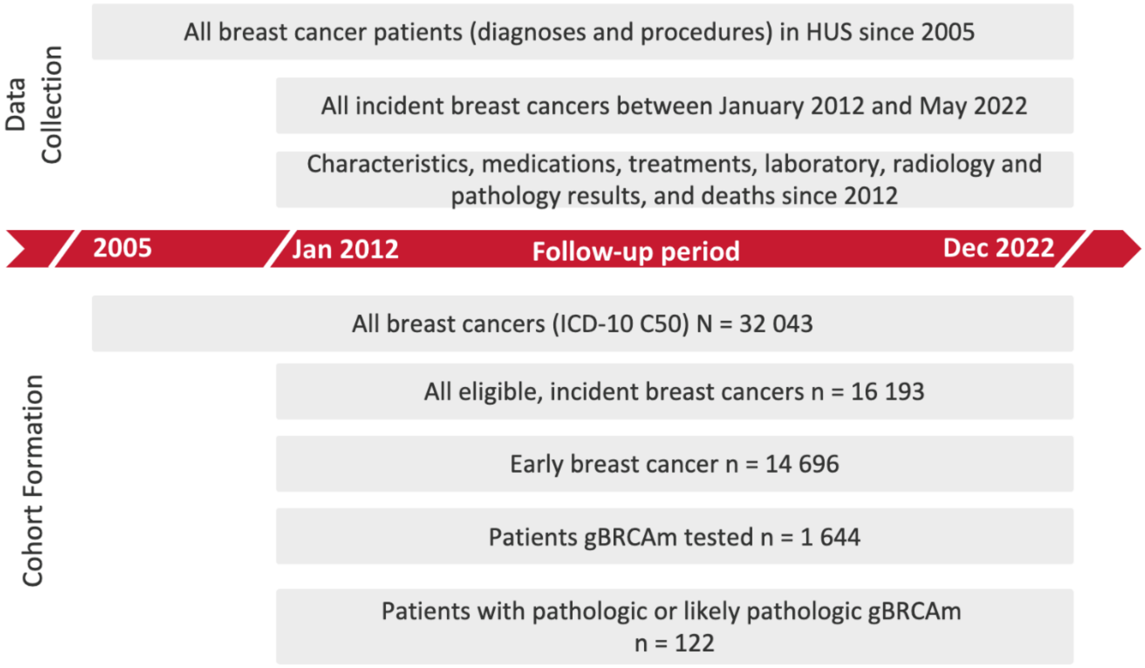

Supplementary Figure 2. Flow chart of the data collection and cohort formation.

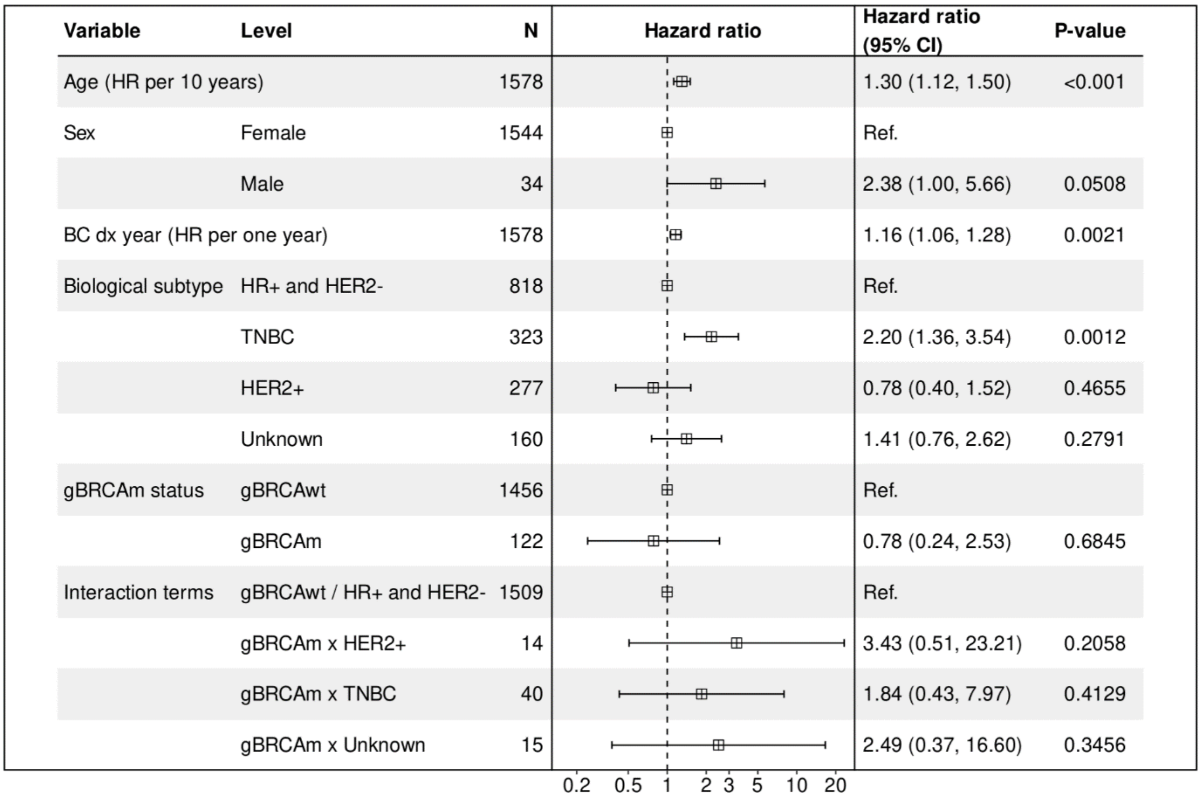

Variable	Level	N	Hazard ratio (95% CI)	P-value
Age (HR per 10 years)		1578	1.30 (1.12, 1.50)	<0.001
Sex	Female	1544	Ref.	
	Male	34	2.38 (1.00, 5.66)	0.0508
BC dx year (HR per one year)		1578	1.16 (1.06, 1.28)	0.0021
Biological subtype	HR+ and HER2-	818	Ref.	
	TNBC	323	2.20 (1.36, 3.54)	0.0012
	HER2+	277	0.78 (0.40, 1.52)	0.4655
	Unknown	160	1.41 (0.76, 2.62)	0.2791
gBRCAm status	gBRCAwt	1456	Ref.	
	gBRCAm	122	0.78 (0.24, 2.53)	0.6845
Interaction terms	gBRCAwt / HR+ and HER2-	1509	Ref.	
	gBRCAm x HER2+	14	3.43 (0.51, 23.21)	0.2058
	gBRCAm x TNBC	40	1.84 (0.43, 7.97)	0.4129
	gBRCAm x Unknown	15	2.49 (0.37, 16.60)	0.3456

Supplementary Figure 3. Cox proportional hazards model for the overall survival (OS) in all pathogenic germline BRCA1/2 mutation (gBRCAm) tested patients.

Abbreviations: BC: breast cancer; dx: diagnosis; HR: hazard ratio; TNBC: triple-negative breast cancer, non-gBRCAm: negative for gBRCAm.

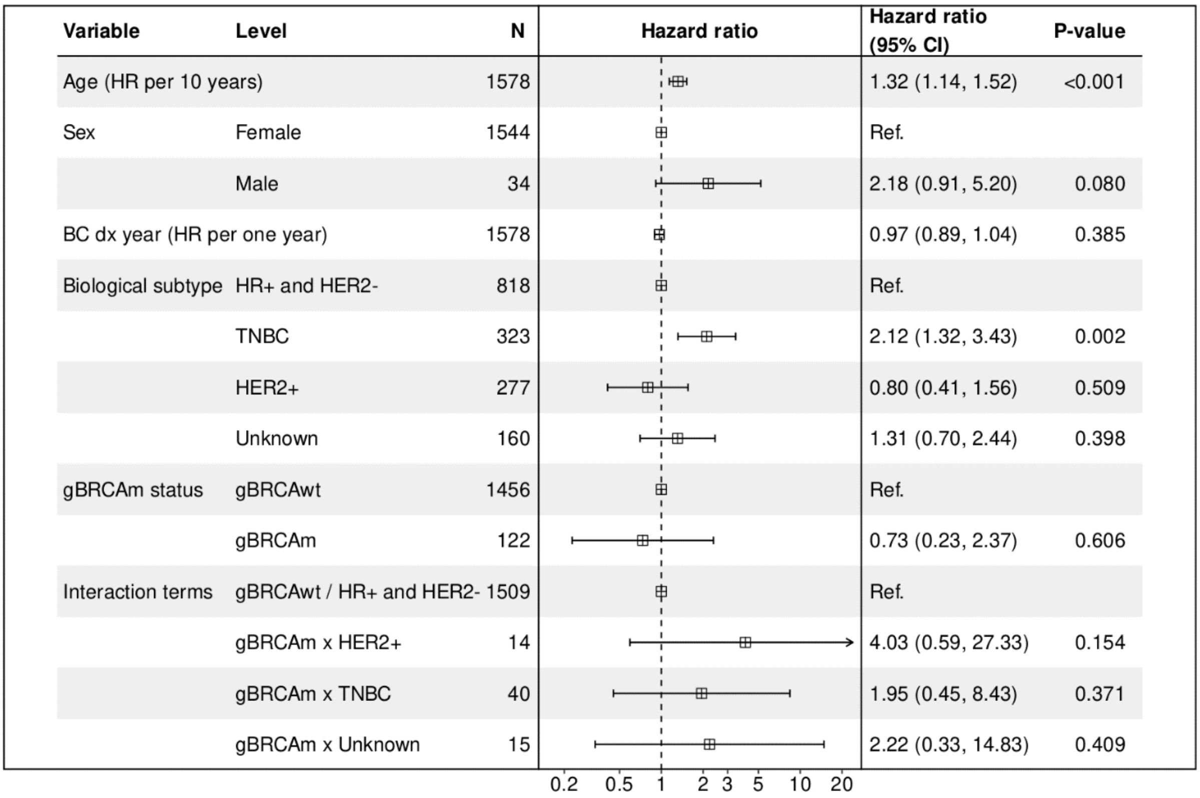

Supplementary Figure 4. Multivariable Cox model for overall survival (OS) defined **as the time from pathogenic germline BRCA1/2 mutation test** (if done before breast cancer diagnosis) until death among all gBRCAm tested patients.

Abbreviations: BC: breast cancer; dx: diagnosis; gBRCAm: pathogenic germline BRCA1/2 mutation; HR: hazard ratio; TNBC: triple-negative breast cancer.

Variable	Level	N	Hazard ratio (95% CI)	P-value
Age (HR per 10 years)		1578	1.10 (0.99, 1.22)	0.063
Sex	Female	1544	Ref.	
	Male	34	1.68 (0.81, 3.49)	0.162
BC dx year (HR per one year)		1578	1.02 (0.97, 1.08)	0.492
Biological subtype	HR+ and HER2-	818	Ref.	
	TNBC	323	1.98 (1.41, 2.76)	<0.001
	HER2+	277	1.47 (1.02, 2.12)	0.039
	Unknown	160	1.22 (0.77, 1.93)	0.397
gBRCAm status	gBRCAwt	1456	Ref.	
	gBRCAm	122	0.82 (0.36, 1.87)	0.634
Interaction terms	gBRCAwt / HR+ and HER2-	1509	Ref.	
	gBRCAm x HER2+	14	2.43 (0.70, 8.43)	0.161
	gBRCAm x TNBC	40	0.98 (0.31, 3.06)	0.970
	gBRCAm x Unknown	15	1.70 (0.39, 7.34)	0.476

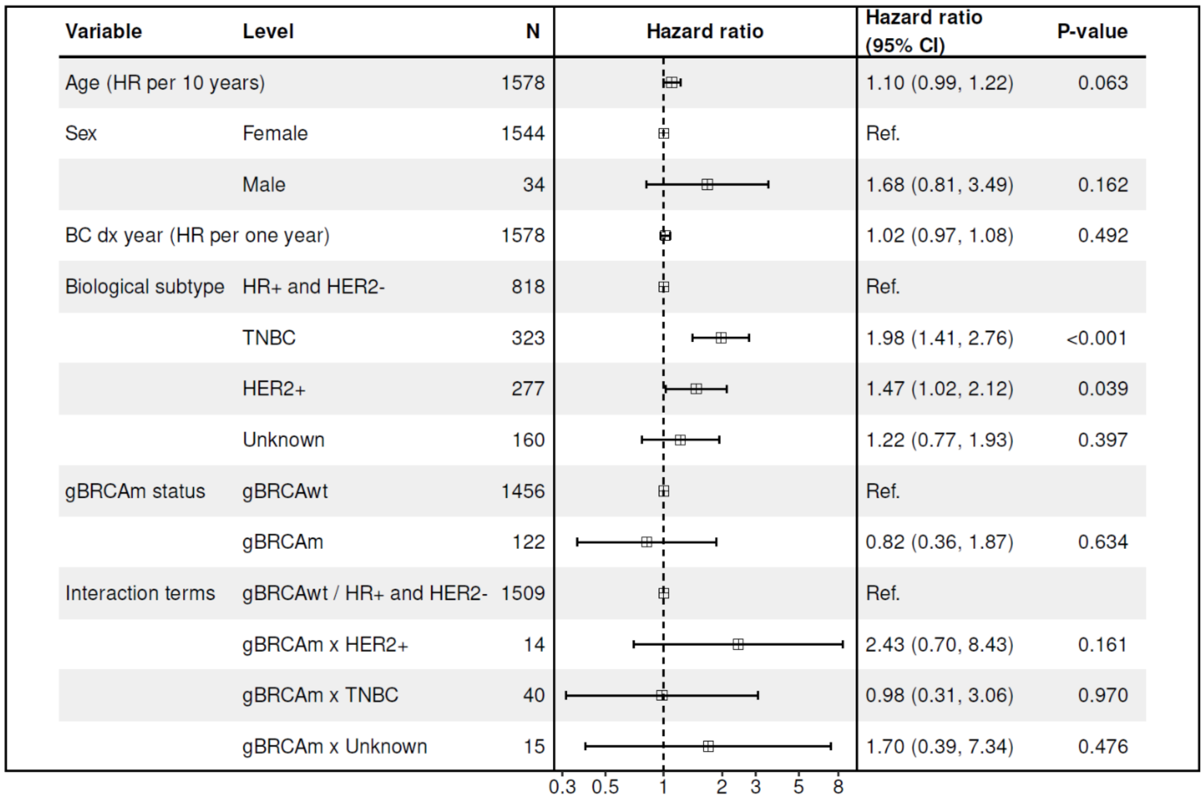

Supplementary Figure 5. Multivariable Cox model for distant disease-free survival (DDFS) among all pathogenic BRCA1/2 mutation (gBRCAm) tested patients.

Abbreviations: BC: breast cancer; dx: diagnosis; HR: hazard ratio; TNBC: triple-negative breast cancer.

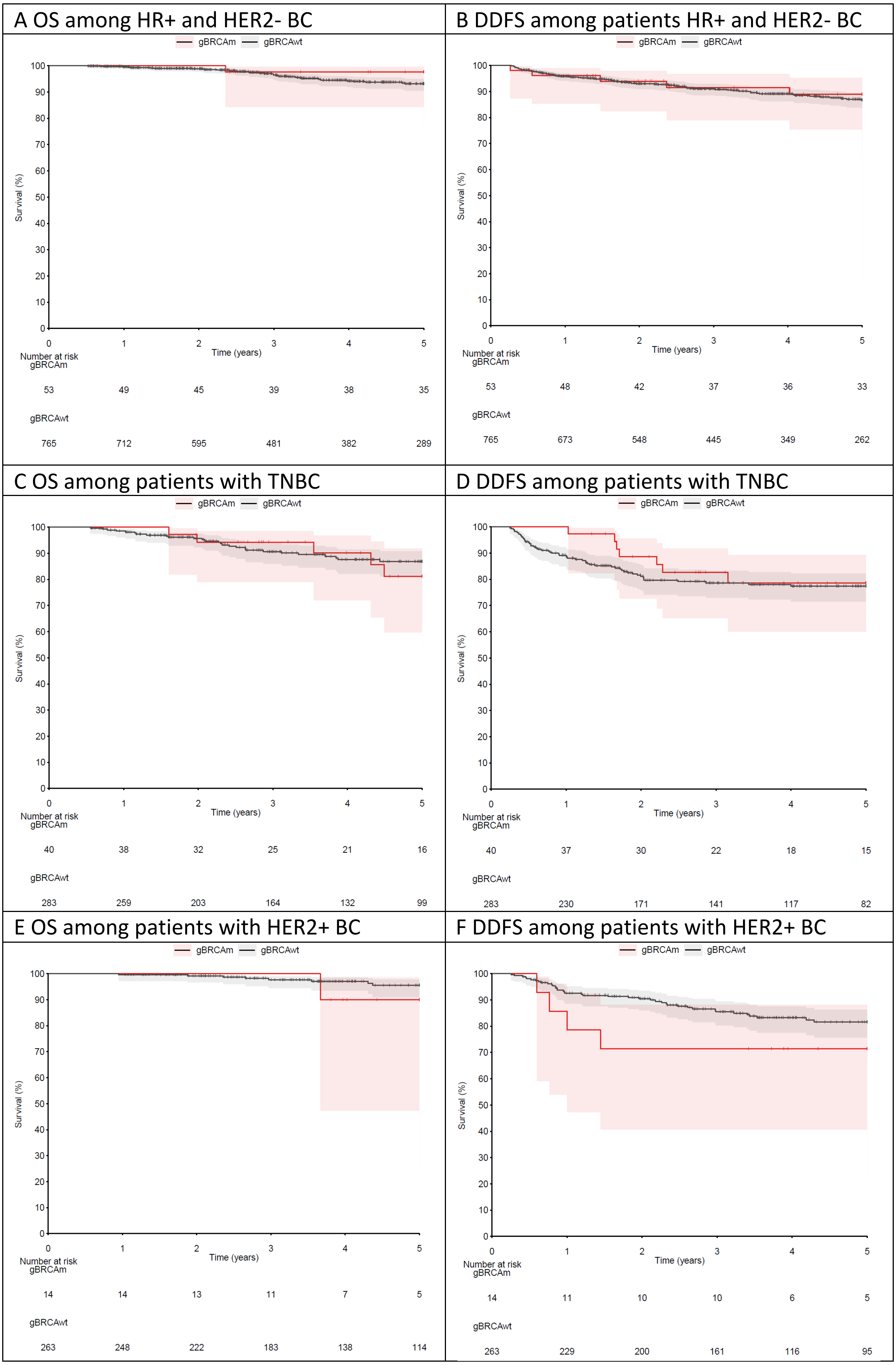
A OS among HR+ and HER2- BC
gBRCAm gBRCAwt
Survival (%)
Time (years)
Number at risk
gBRCAm
53 49 45 39 38 35
gBRCAwt
765 712 595 481 382 289
B DDFS among patients HR+ and HER2- BC
gBRCAm gBRCAwt
Survival (%)
Time (years)
Number at risk
gBRCAm
53 48 42 37 36 33
gBRCAwt
765 673 548 445 349 262
C OS among patients with TNBC
gBRCAm gBRCAwt
Survival (%)
Time (years)
Number at risk
gBRCAm
40 38 32 25 21 16
gBRCAwt
283 259 203 164 132 99
D DDFS among patients with TNBC
gBRCAm gBRCAwt
Survival (%)
Time (years)
Number at risk
gBRCAm
40 37 30 22 18 15
gBRCAwt
283 230 171 141 117 82
E OS among patients with HER2+ BC
gBRCAm gBRCAwt
Survival (%)
Time (years)
Number at risk
gBRCAm
14 14 13 11 7 5
gBRCAwt
263 248 222 183 138 114
F DDFS among patients with HER2+ BC
gBRCAm gBRCAwt
Survival (%)
Time (years)
Number at risk
gBRCAm
14 11 10 10 6 5
gBRCAwt
263 229 200 161 116 95

Supplementary Figure 6. A) Overall survival (OS), and B) distant disease-free survival (DDFS) among patients with HR+ and HER- breast cancer separately; C) OS and D) DDFS in patients with TNBC breast cancer; E) OS and F) DDFS among patients with HER2+ breast cancer. The results were analysed separately among patients with (gBRCAm) and without pathogenic gBRCA1/2 mutation (gBRCAwt). The shaded areas correspond to 95% confidence intervals (CIs) of the Kaplan-Meier estimates.

Abbreviations: HR: hormone receptor; OS: overall survival; DDFS: distant disease-free survival; TNBC: triple-negative breast cancer.

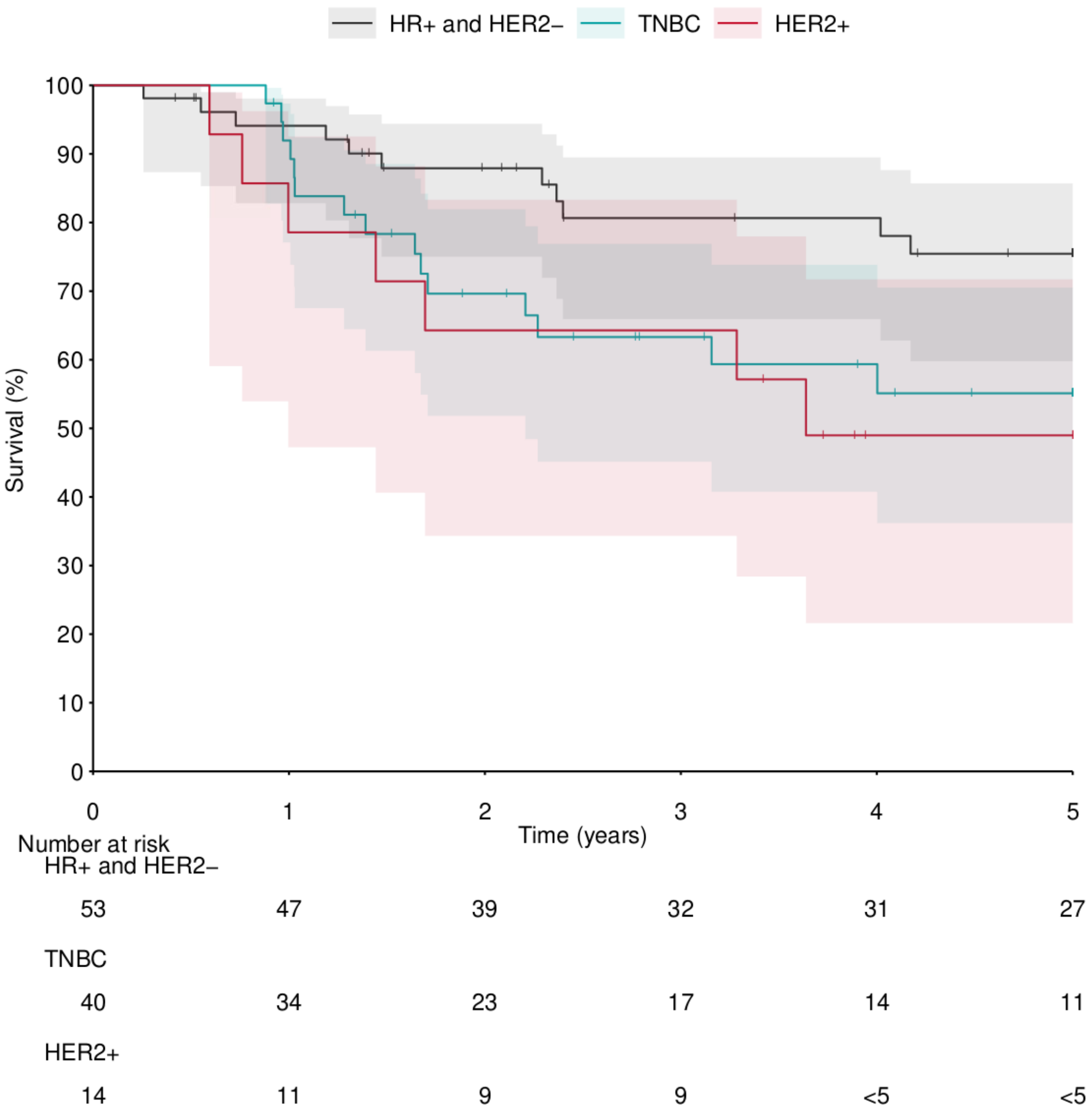

Supplementary Figure 7 Invasive disease-free survival among patients with pathogenic gBRCA1/2 mutation according to the biological subgroup of the breast cancer.

Abbreviations: HR: hormone receptor; TNBC: triple-negative breast cancer.

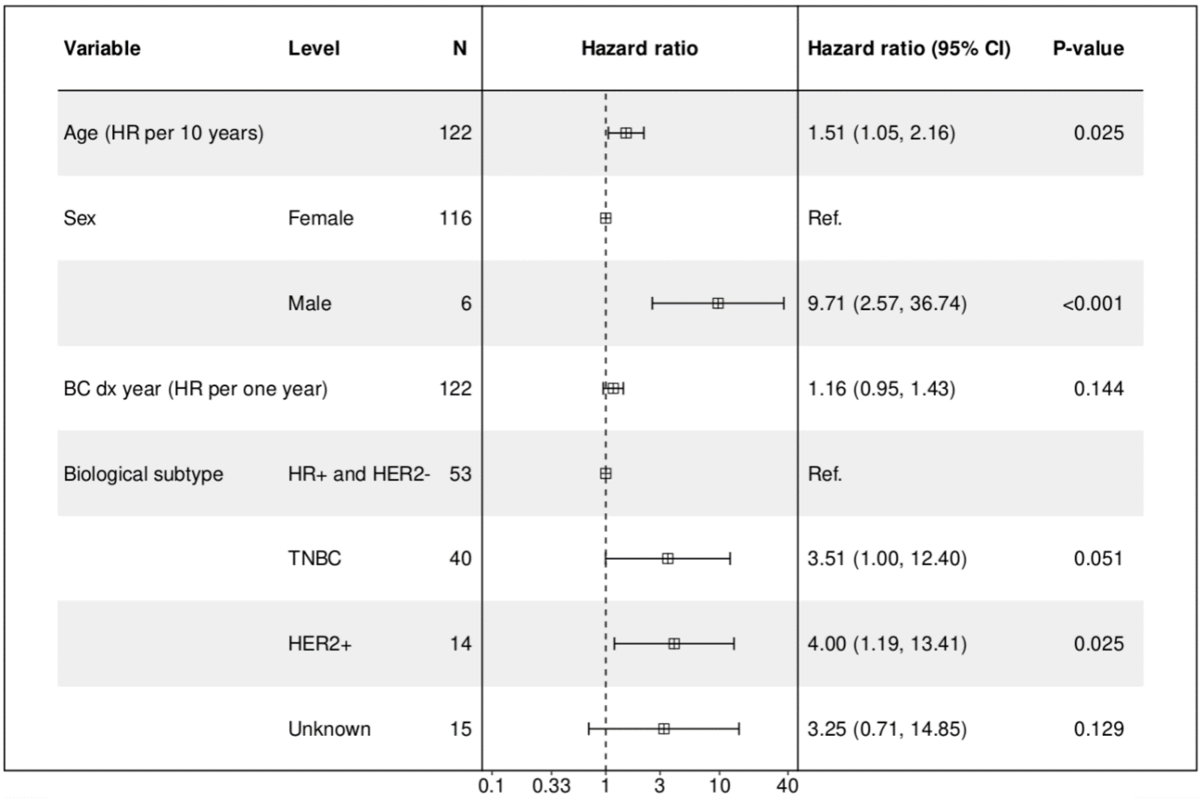

Supplementary Figure 8. Multivariable Cox model for distant disease-free survival (DDFS) among patients with pathogenic germline BRCA1/2 mutation (gBRCAm) early breast cancer.

Abbreviations: BC: breast cancer; dx: diagnosis; HR: hazard ratio; TNBC: triple-negative breast cancer.

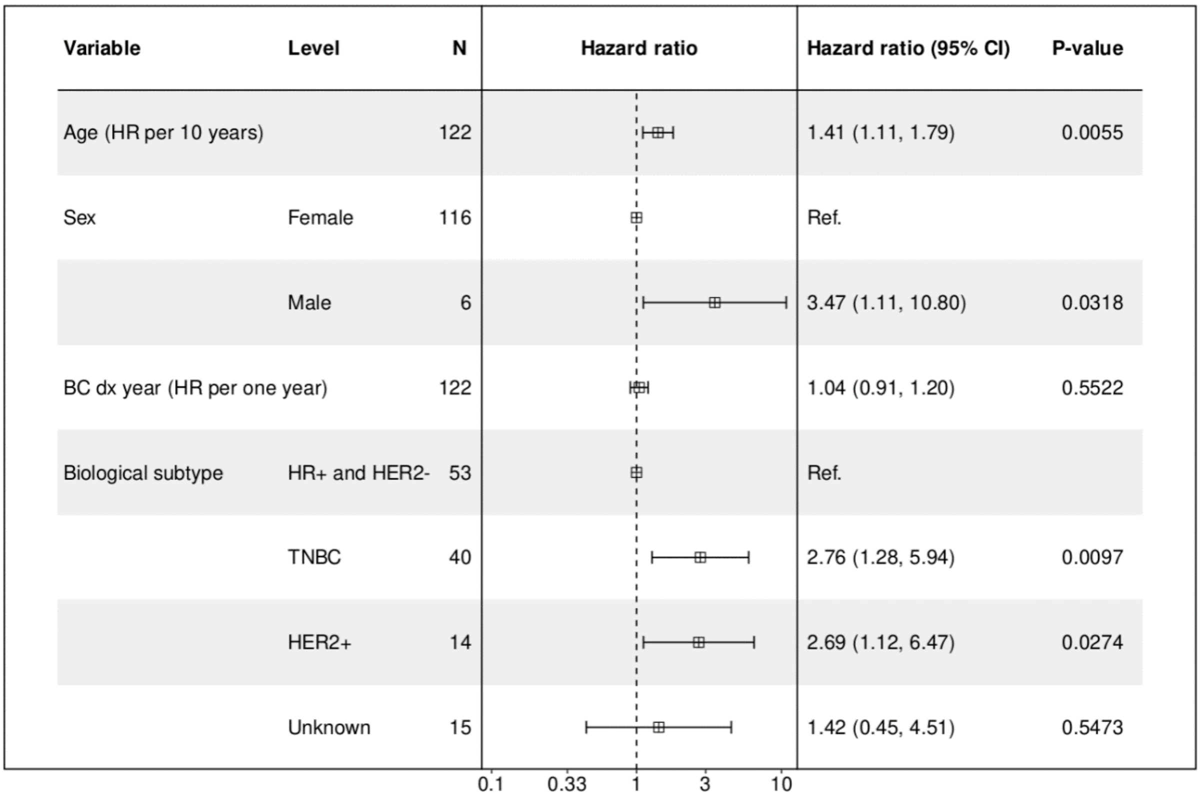

Supplementary Figure 9. Multivariable Cox model for invasive disease-free survival (IDFS) among patients with pathogenic germline BRCA1/2 mutation (gBRCAm) early-stage breast cancer.

Abbreviations: BC: breast cancer; dx: diagnosis; HR: hazard ratio; TNBC = triple-negative breast cancer.

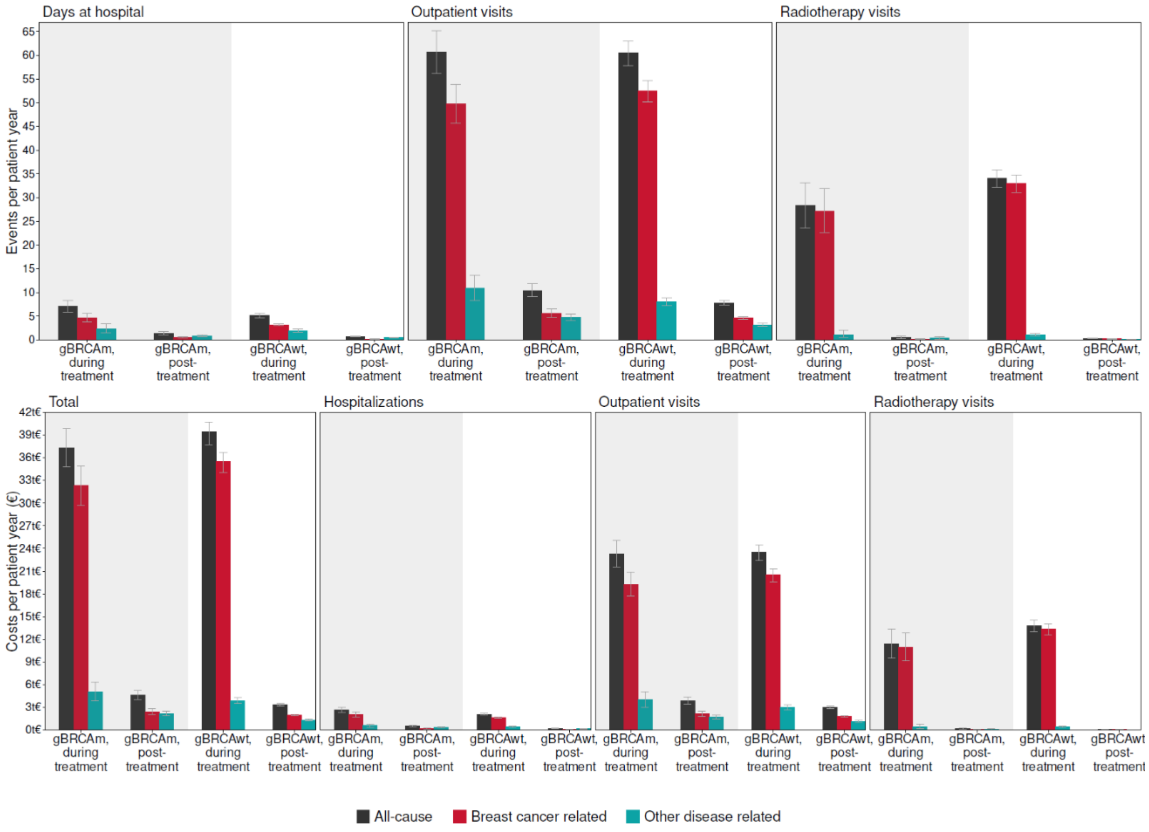

Supplementary Figure 10: Healthcare resource utilization (HCRU) and related costs per patient year in the patients with (gBRCAm, shaded area) and without (gBRCAwt, unshaded area) pathogenic germline BRCA1/2 mutation during adjuvant treatment (time from breast cancer diagnosis until the end of adjuvant chemotherapy or radiotherapy) and during follow-up afterwards. The all-cause results were reported as well as breast cancer related and other disease related HCRU and costs.

Supplementary Table 1. Patient demographics and cancer characteristics of patients with pathogenic germline BRCA1/2 mutation (gBRCAm) and early-stage breast cancer according to the biological subgroup.

		**HER2+**		**HR+ and HER2-**		**TNBC**	
**Variable**	**Level**	**N**	**%**	**N**	**%**	**N**	**%**
**N**	-	14	100	53	100	40	100
**Sex**	Female	Censored*	Censored*	48	90.6	40	100
	Male	< 5	-	5	9.4	0	0
**Age at breast cancer diagnosis (years)****	Median (IQR)	53.9 (43.9-62)		48.8 (41.9-60.2)		40.3 (34.8-51.5)	
**Age group at breast cancer diagnosis (years)**	Less than 45	5	35.7	19	35.8	24	60
	45 - 54	< 5	-	16	30.2	12	30
	55 - 64	< 5	-	9	17	< 5	-
	65 +	< 5	-	9	17	< 5	-
**Number of positive lymph nodes[1]**	0	< 5	-	19	42.2	16	61.5
	1-3	< 5	-	17	37.8	Censored*	Censored*
	4+	< 5	-	9	20	< 5	-
**Stage[2]**	I	< 5	-	11	26.8	10	41.7
	II	< 5	-	20	48.8	Censored*	Censored*
	III	< 5	-	10	24.4	< 5	-
**Grade[3]**	1-2	Censored*	Censored*	26	53.1	Censored*	Censored*
	3	< 5	-	Censored*	Censored*	< 5	-
**IHC score (HER2)[3]**	0	0	0	8	18.6	18	66.7
	1+	0	0	8	18.6	< 5	-
	2+	10	83.3	26	60.5	8	29.6
	3+	< 5	-	< 5	-	0	0
**Charlson comorbidity index (Quan et al)**	0	12	85.7	47	88.7	37	92.5
	1 +	< 5	-	6	11.3	< 5	-
**Proliferation (Ki-67)[4]**	Low (less than 20%)	< 5	-	8	22.2	< 5	-
	High (at least 20%)	10	90.9	28	77.8	20	87
**Tumour size[5]**	At most 2cm	8	57.1	22	46.8	22	71
	More than 2cm	6	42.9	25	53.2	9	29
**Length of follow-up (years)**	Median (IQR)	4 (3.5-5.7)		6 (2.5-7.5)		4.2 (2.6-6.7)	
	Mean (SD)	4.7 (2.3)		5.5 (2.9)		4.5 (2.4)	

**Per Finnish legislation, groups with patient numbers 1-4 cannot be reported, and the exact number of patients may not be inferable. Thus, at least one other (sub)group within the category must be censored to prevent retrieving the exact number of patients in all subgroups.*

***P-value for the difference in median values from* the Kruskal-*Wallis test: 0.002.*

[1]Missing values: HER2+: < 5, HR+ and HER2- 8 (15.1%), TNBC 14 (35%).
[2] Missing values: HER2+: < 5, HR+ and HER2- 12 (22.6%), TNBC 16 (40%).
[3] Missing values: HER2+: <5, HR+ and HER2- censored, TNBC censored.
[4] Missing values: HER2+: < 5, HR+ and HER2- 17 (32.1%), TNBC censored.
[5] Missing values: HER2+: 0, HR+ and HER2- 6 (11.3%), TNBC 9 (22.5%).

Abbreviations: IHC:immunohistochemistry. IQR: interquartile range. SD: standard deviation.

Supplementary Table 2: Numerical estimates at one year interval up to five years of all outcome analyses.

Outcome	Subgroup	Time (years)	Number at risk	Estimate (%)	Lower 95% CI	Upper 95% CI
OS	gBRCAm	1	113	99.1	93.9	99.9
OS	gBRCAm	2	100	96.3	90.4	98.6
OS	gBRCAm	3	85	95.3	89.0	98.0
OS	gBRCAm	4	72	92.9	85.5	96.6
OS	gBRCAm	5	62	90.1	81.7	94.8
OS	gBRCAwt	1	1353	99.4	98.9	99.7
OS	gBRCAwt	2	1140	98.3	97.4	98.8
OS	gBRCAwt	3	923	95.4	94.1	96.5
OS	gBRCAwt	4	731	93.4	91.7	94.7
OS	gBRCAwt	5	570	92.4	90.5	93.9
IDFS	gBRCAm	1	102	89.7	82.6	94.0
IDFS	gBRCAm	2	79	77.1	68.1	83.8
IDFS	gBRCAm	3	66	72.0	62.5	79.4
IDFS	gBRCAm	4	53	68.6	58.7	76.5
IDFS	gBRCAm	5	46	64.6	54.4	73.2
DDFS	BRCAmut	1	107	94.0	87.9	97.1
DDFS	BRCAmut	2	91	87.6	80.0	92.5
DDFS	BRCAmut	3	78	84.6	76.4	90.2
DDFS	BRCAmut	4	66	83.5	75.1	89.3
DDFS	BRCAmut	5	59	82.3	73.5	88.4
DDFS	gBRCAm	1	107	94.0	87.9	97.1
DDFS	gBRCAm	2	91	87.6	80.0	92.5
DDFS	gBRCAm	3	78	84.6	76.4	90.2
DDFS	gBRCAm	4	66	83.5	75.1	89.3
DDFS	gBRCAm	5	59	82.3	73.5	88.4
DDFS	gBRCAwt	1	1259	94.0	92.6	95.1
DDFS	gBRCAwt	2	1029	90.2	88.4	91.6
DDFS	gBRCAwt	3	834	87.4	85.5	89.2
DDFS	gBRCAwt	4	656	85.5	83.3	87.4
DDFS	gBRCAwt	5	498	83.5	81.1	85.6
OS	gBRCAm	1	14	100.0	100.0	100.0
OS	gBRCAm	1	49	100.0	NA	NA
OS	gBRCAm	1	38	100.0	NA	NA
OS	gBRCAm	2	13	100.0	NA	NA
OS	gBRCAm	2	45	100.0	NA	NA

OS	gBRCAm	2	32	94.3	79.0	98.5
OS	gBRCAm	3	11	100.0	NA	NA
OS	gBRCAm	3	39	97.6	84.3	99.7
OS	gBRCAm	3	25	94.3	79.0	98.5
OS	gBRCAm	4	7	90.0	47.3	98.5
OS	gBRCAm	4	38	97.6	84.3	99.7
OS	gBRCAm	4	21	90.2	72.0	96.8
OS	gBRCAm	5	5	90.0	47.3	98.5
OS	gBRCAm	5	35	97.6	84.3	99.7
OS	gBRCAm	5	16	81.2	59.7	91.9
OS	gBRCAwt	1	248	99.6	97.2	99.9
OS	gBRCAwt	1	712	99.6	98.8	99.9
OS	gBRCAwt	1	259	98.5	96.1	99.4
OS	gBRCAwt	2	222	99.2	96.7	99.8
OS	gBRCAwt	2	595	98.9	97.7	99.4
OS	gBRCAwt	2	203	95.7	92.3	97.6
OS	gBRCAwt	3	183	97.7	94.4	99.0
OS	gBRCAwt	3	481	96.4	94.6	97.7
OS	gBRCAwt	3	164	90.7	86.0	93.8
OS	gBRCAwt	4	138	97.1	93.5	98.7
OS	gBRCAwt	4	382	94.3	91.9	95.9
OS	gBRCAwt	4	132	87.6	82.1	91.4
OS	gBRCAwt	5	114	95.5	91.0	97.8
OS	gBRCAwt	5	289	93.1	90.5	95.1
OS	gBRCAwt	5	99	86.8	81.2	90.9
DDFS	gBRCAm	1	11	78.6	47.2	92.5
DDFS	gBRCAm	1	48	96.1	85.3	99.0
DDFS	gBRCAm	1	37	100.0	NA	NA
DDFS	gBRCAm	2	10	71.4	40.6	88.2
DDFS	gBRCAm	2	42	94.0	82.4	98.0
DDFS	gBRCAm	2	30	88.7	72.6	95.6
DDFS	gBRCAm	3	10	71.4	40.6	88.2
DDFS	gBRCAm	3	37	91.6	78.9	96.8
DDFS	gBRCAm	3	22	82.6	65.2	91.8
DDFS	gBRCAm	4	6	71.4	40.6	88.2
DDFS	gBRCAm	4	36	91.6	78.9	96.8
DDFS	gBRCAm	4	18	78.7	60.0	89.3
DDFS	gBRCAm	5	5	71.4	40.6	88.2
DDFS	gBRCAm	5	33	89.0	75.4	95.3

DDFS	gBRCAm	5	15	78.7	60.0	89.3
DDFS	gBRCAwt	1	229	92.5	88.6	95.2
DDFS	gBRCAwt	1	673	95.9	94.2	97.1
DDFS	gBRCAwt	1	230	89.2	84.9	92.3
DDFS	gBRCAwt	2	200	90.4	86.0	93.5
DDFS	gBRCAwt	2	548	93.1	90.9	94.7
DDFS	gBRCAwt	2	171	81.1	75.8	85.4
DDFS	gBRCAwt	3	161	85.5	80.3	89.5
DDFS	gBRCAwt	3	445	91.0	88.6	93.0
DDFS	gBRCAwt	3	141	78.7	73.0	83.3
DDFS	gBRCAwt	4	116	83.2	77.6	87.6
DDFS	gBRCAwt	4	349	89.0	86.3	91.3
DDFS	gBRCAwt	4	117	77.4	71.5	82.2
DDFS	gBRCAwt	5	95	81.7	75.6	86.3
DDFS	gBRCAwt	5	262	86.7	83.5	89.3
DDFS	gBRCAwt	5	82	77.4	71.5	82.2
IDFS	HER2+	1	11	78.6	47.2	92.5
IDFS	HER2+	2	9	64.3	34.3	83.3
IDFS	HER2+	3	9	64.3	34.3	83.3
IDFS	HR+ and HER2-	1	47	94.1	82.8	98.1
IDFS	HR+ and HER2-	2	39	87.9	75.0	94.4
IDFS	HR+ and HER2-	3	32	80.7	65.9	89.5
IDFS	HR+ and HER2-	4	31	80.7	65.9	89.5
IDFS	HR+ and HER2-	5	27	75.4	59.8	85.7
IDFS	TNBC	1	34	92.0	77.1	97.3
IDFS	TNBC	2	23	69.6	51.8	81.9
IDFS	TNBC	3	17	63.3	45.1	76.9
IDFS	TNBC	4	14	59.4	40.8	73.8
IDFS	TNBC	5	11	55.1	36.2	70.5

Supplementary Table 3. Numerical estimates of the health care resource utilization and related costs per patient year among patients without pathogenic gBRCA1/2 mutation according to the biological subgroup of the breast cancer.

			Events (N) per patient year			Cost (€; 2022 prices) per patient year		
**Type**	**Subgroup**	**Specificity**	**Avg.**	**Lower 95% CI**	**Upper 95% CI**	**Avg.**	**Lower 95% CI**	**Upper 95% CI**
**Hospitalizations**	HER2+	All-cause	0.5	0.5	0.6	572.63	509.11	641.00
**Hospitalizations**	HR+ and HER2-	All-cause	0.4	0.4	0.5	490.45	454.19	528.98
**Hospitalizations**	TNBC	All-cause	0.5	0.4	0.5	491.71	435.58	553.39
**Hospitalizations**	Unknown	All-cause	0.4	0.3	0.4	381.67	300.80	468.30
**Inpatient**	HER2+	All-cause	1.5	1.3	1.7	-	-	-
**Inpatient**	HR+ and HER2-	All-cause	1.3	1.2	1.4	-	-	-
**Inpatient**	TNBC	All-cause	1.5	1.3	1.9	-	-	-
**Inpatient**	Unknown	All-cause	1.1	0.8	1.4	-	-	-
**Outpatient clinic contacts**	HER2+	All-cause	18.9	17.5	20.3	7323.41	6781.28	7910.22
**Outpatient clinic contacts**	HR+ and HER2-	All-cause	12.9	12.2	13.6	4890.44	4621.45	5172.97
**Outpatient clinic contacts**	TNBC	All-cause	15.3	14.0	16.8	5924.46	5419.67	6516.71
**Outpatient clinic contacts**	Unknown	All-cause	8.1	7.1	9.3	3005.52	2617.00	3446.52
**Radiotherapy**	HER2+	All-cause	4.4	3.9	4.8	1749.35	1580.61	1920.52
**Radiotherapy**	HR+ and HER2-	All-cause	3.5	3.3	3.8	1435.71	1349.41	1520.41
**Radiotherapy**	TNBC	All-cause	4.6	4.2	5.0	1840.02	1680.06	2008.21
**Radiotherapy**	Unknown	All-cause	1.6	1.2	2.1	650.45	481.04	837.36
**Total**	HER2+	All-cause	-	-	-	9645.39	9013.41	10337.35
**Total**	HR+ and HER2-	All-cause	-	-	-	6816.60	6501.07	7145.56
**Total**	TNBC	All-cause	-	-	-	8256.19	7670.62	8927.81
**Total**	Unknown	All-cause	-	-	-	4037.65	3536.29	4589.04
**Hospitalizations**	HER2+	BC	0.3	0.3	0.4	381.71	335.80	429.36
**Hospitalizations**	HR+ and HER2-	BC	0.3	0.2	0.3	308.15	283.64	333.65
**Hospitalizations**	TNBC	BC	0.2	0.2	0.3	286.92	248.26	330.27
**Hospitalizations**	Unknown	BC	0.1	0.1	0.1	132.81	95.82	173.01
**Inpatient**	HER2+	BC	0.8	0.7	1.0	-	-	-
**Inpatient**	HR+ and HER2-	BC	0.7	0.6	0.7	-	-	-
**Inpatient**	TNBC	BC	0.6	0.5	0.7	-	-	-

**Inpatient**	Unknown	BC	0.3	0.2	0.4	-	-	-
**Outpatient clinic contacts**	HER2+	BC	14.7	13.6	15.9	5772.23	5345.51	6248.17
**Outpatient clinic contacts**	HR+ and HER2-	BC	9.1	8.6	9.6	3522.45	3336.74	3715.33
**Outpatient clinic contacts**	TNBC	BC	11.5	10.5	12.7	4513.37	4109.46	4989.19
**Outpatient clinic contacts**	Unknown	BC	4.3	3.5	5.3	1668.53	1337.85	2051.03
**Radiotherapy**	HER2+	BC	4.2	3.8	4.6	1685.55	1517.58	1856.73
**Radiotherapy**	HR+ and HER2-	BC	3.4	3.2	3.6	1373.20	1288.95	1456.59
**Radiotherapy**	TNBC	BC	4.4	4.1	4.8	1791.86	1637.23	1952.69
**Radiotherapy**	Unknown	BC	1.3	0.9	1.7	521.03	364.29	696.56
**Total**	HER2+	BC	-	-	-	7839.48	7312.81	8417.68
**Total**	HR+ and HER2-	BC	-	-	-	5203.79	4969.35	5438.79
**Total**	TNBC	BC	-	-	-	6592.15	6110.24	7146.26
**Total**	Unknown	BC	-	-	-	2322.37	1876.94	2827.28
**Hospitalizations**	HER2+	Other	0.2	0.2	0.2	190.93	155.20	229.34
**Hospitalizations**	HR+ and HER2-	Other	0.2	0.2	0.2	182.30	159.54	207.02
**Hospitalizations**	TNBC	Other	0.2	0.2	0.3	204.79	164.99	248.92
**Hospitalizations**	Unknown	Other	0.3	0.2	0.3	248.86	179.49	325.62
**Inpatient**	HER2+	Other	0.7	0.5	0.8	-	-	-
**Inpatient**	HR+ and HER2-	Other	0.7	0.6	0.8	-	-	-
**Inpatient**	TNBC	Other	0.9	0.7	1.2	-	-	-
**Inpatient**	Unknown	Other	0.8	0.5	1.0	-	-	-
**Outpatient clinic contacts**	HER2+	Other	4.2	3.6	4.8	1551.18	1346.73	1769.59
**Outpatient clinic contacts**	HR+ and HER2-	Other	3.8	3.4	4.2	1368.00	1223.12	1537.39
**Outpatient clinic contacts**	TNBC	Other	3.8	3.4	4.3	1411.09	1238.51	1610.21
**Outpatient clinic contacts**	Unknown	Other	3.8	3.3	4.4	1336.99	1145.28	1539.55
**Radiotherapy**	HER2+	Other	0.2	0.1	0.3	63.80	26.68	111.52
**Radiotherapy**	HR+ and HER2-	Other	0.2	0.1	0.2	62.51	42.98	85.24
**Radiotherapy**	TNBC	Other	0.1	0.1	0.2	48.16	24.42	85.33
**Radiotherapy**	Unknown	Other	0.3	0.1	0.6	129.42	54.87	220.69
**Total**	HER2+	Other	-	-	-	1805.91	1570.49	2060.97
**Total**	HR+ and HER2-	Other	-	-	-	1612.81	1454.60	1792.68
**Total**	TNBC	Other	-	-	-	1664.04	1467.53	1894.83

**Total**	Unknown	Other	-	-	-	1715.27	1462.14	1981.07
